# Intestinal mucin activates human dendritic cells and IL-8 production in a glycan-specific manner

**DOI:** 10.1074/jbc.M117.789305

**Published:** 2018-03-26

**Authors:** Felipe Melo-Gonzalez, Thomas M. Fenton, Cecilia Forss, Catherine Smedley, Anu Goenka, Andrew S. MacDonald, David J. Thornton, Mark A. Travis

**Affiliations:** From the ‡ Manchester Collaborative Centre for Inflammation Research,; the § Wellcome Trust Centre for Cell-Matrix Research, and; the ¶ Manchester Immunology Group, Faculty of Biology, Medicine and Health, Manchester Academic Health Sciences Centre, University of Manchester, Manchester M13 9NT, United Kingdom

**Keywords:** dendritic cell, inflammation, intestine, mucin, mucosal immunology

## Abstract

Cross-talk between different components of the intestinal barrier and the immune system may be important in maintaining gut homeostasis. A crucial part of the gut barrier is the mucus layer, a cross-linked gel on top of the intestinal epithelium that consists predominantly of the mucin glycoprotein MUC2. However, whether the mucin layer actively regulates intestinal immune cell responses is not clear. Because recent evidence suggests that intestinal dendritic cells (DCs) may be regulated by the mucus layer, we purified intestinal mucin, incubated it with human DCs, and determined the functional effects. Here we show that expression of the chemokine IL-8 and co-stimulatory DC markers CD86 and CD83 are significantly up-regulated on human DCs in the presence of intestinal mucins. Additionally, mucin-exposed DCs promoted neutrophil migration in an IL-8–dependent manner. The stimulatory effects of mucins on DCs were not due to mucin sample contaminants such as lipopolysaccharide, DNA, or contaminant proteins. Instead, mucin glycans are important for the pro-inflammatory effects on DCs. Thus, intestinal mucins are capable of inducing important pro-inflammatory functions in DCs, which could be important in driving inflammatory responses upon intestinal barrier damage.

## Introduction

The intestine is a difficult environment for the immune system, which must act to promote responses to ingested pathogens but remain tolerant against the multitude of microorganisms that constitute the intestinal microbiota. The intestinal mucus layer, a dynamic, viscoelastic fluid, acts as an important barrier to separate the intestinal tissue and immune system from the microbiota and contents of the lumen and also plays an important defensive role against pathogens (1–4). Mucus is present along the entire length of the intestine, with a discontinuous layer present in the small intestine and a thicker, two-layered structure in the large intestine: an inner layer that is firmly attached to the epithelium and a looser outer layer (3, 5). The main structural component of intestinal mucus is the goblet cell-secreted mucin MUC2 (6), which in the inner mucus layer forms a dense network of cross-linked polymers, acting as an impenetrable barrier to enteric bacteria. In contrast, MUC2 forms an expanded structure in the outer mucus layer caused by partial proteolytic cleavage of the mucin, which allows components of the microbiota to penetrate and reside within the mucus (5).

Because the mucus layer sits directly on top of the intestinal epithelial cells, this barrier is likely to be in close contact with cells of the immune system that are present within or just below the epithelial layer. For example, dendritic cells (DCs)^4^ within intestinal tissue are proposed to continuously sample luminal antigens and are essential in maintaining gut homeostasis (7). Recent data have suggested that MUC2 can directly act upon DCs to suppress their activation in the presence of inflammatory stimuli, instead promoting anti-inflammatory responses (8), and that intestinal DC proportions are altered in mice lacking expression of Muc2 (9). Thus, how mucins act to regulate DC function requires further attention.

To this end, density gradient centrifugation was employed to purify mucins from a human colonic cell line and from both mouse small and large intestine. Following detailed biochemical characterization of mucin preparations, we examined the effect of intestinal mucin on human monocyte-derived DCs (moDCs). Surprisingly, in contrast to previous reports, we found that both human mucin and murine intestinal mucin induced activation of human DCs, showing enhanced expression of the co-stimulatory molecules CD83 and CD86, and enhanced production of the pro-inflammatory chemokine IL-8. Such activation was not a result of bacterial or protein contaminants but instead depended on glycosylation of the mucin. After mucin treatment, DCs were able to promote neutrophil migration in an IL-8–dependent fashion. Thus, our results suggest that mucins can have pro-inflammatory properties on DCs, which may be important in regulation of intestinal immune responses during homeostasis and barrier disruption.

## Results

### Human and mouse intestinal mucins induce IL-8 expression by moDCs

To study the interaction between intestinal mucins and DC, we first investigated the role of purified human mucins on modulation of DC function. Thus, we purified mucin from the LS174T colonic cell line, which has previously been shown to express high levels of the mucin MUC2, the major secreted mucin found in intestinal mucus (6, 10). MUC2 was purified from conditioned cell culture supernatants by CsCl/GuHCl isopycnic density gradient centrifugation, which separates mucins from other macromolecules present. Sample composition was confirmed by MS and mucin size and concentration determined by size-exclusion chromatography–multiangle laser light scattering (SEC–MALLS).

**Figure 1.**
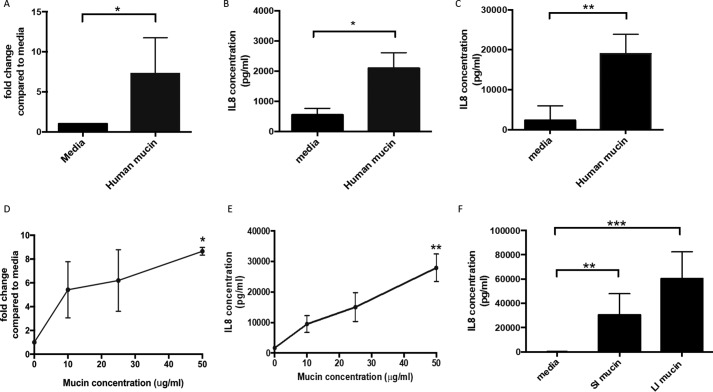
**Human and mouse intestinal mucin can promote IL-8 production on moDCs.** Human moDCs were treated overnight with human secreted mucin (2 or 50 μg/ml) and different concentrations of mouse large intestinal mucin (10, 25, and 50 μg/ml). IL-8 expression was measured by qPCR and ELISA. *A* and *B*, moDCs were stimulated overnight with human secreted mucin (2 μg/ml) and IL-8 expression measured by qPCR (*A*) and ELISA (*B*). *n* ≥ 5 independent experiments; *, *p* < 0.05 assessed using unpaired Student's *t* test. *C*, ELISA was performed to detect IL-8 in supernatants from untreated and human secreted mucin (50 μg/ml)-treated moDCs. *n* = 6 independent experiments; **, *p* < 0.01 assessed using unpaired Student's *t* test. *D* and *E*, moDCs were treated with increasing concentrations of mouse large intestinal mucin (10, 25, and 50 μg/ml) and IL-8 measured by qPCR (*D*, *n* = 3; *, *p* < 0.05 assessed using one-way ANOVA with Dunnet's multiple comparison test) and ELISA (*E*, *n* = 5; **, *p* < 0.01 assessed via Kruskal–Wallis test with Dunn's multiple comparison test). *F*, moDCs were stimulated overnight with mouse small intestinal (*SI*) and large intestinal (*LI*) mucins (50 μg/ml both). ELISA was performed to detect IL-8 on supernatants from untreated and mucin-treated moDCs. *n* = 8. **, *p* < 0.01; ***, *p* < 0.001 assessed using Kruskal–Wallis test with Dunn's multiple comparison test.

To determine a potential role for human secreted mucins in the modulation of DC function, human moDCs were treated with purified mucin MUC2. The phenotype of human moDCs was confirmed by flow cytometry, with cells showing a lack of CD14 expression and reduced expression of CD33 compared with monocytes and high expression of the DC-associated markers HLA-DR, CD11c, CD1c, CD11b, CD141, and CD13 (Fig. S1). Changes in moDC gene expression after incubation with a preparation enriched in human MUC2 were analyzed using a DC PCR array, which analyses expression of 84 genes related to DC and antigen presenting cell function. Interestingly, a number of DC-related genes were up-regulated, with one of the most highly up-regulated genes stimulated by mucin treatment being IL-8 (Table S1), an important chemokine in the recruitment of innate cells to infection sites (11). Up-regulation of IL-8 by human mucin treatment was confirmed by qPCR and ELISA (Fig. 1, *A* and *B*), with both low (2 μg/ml) and high (50 μg/ml) mucin doses showing similar effects (Fig. 1, *A–C*). Therefore, these results suggest that IL-8 production by human moDCs may be induced by interactions with human secreted MUC2.

Because LS174T cells are a transformed cell line, mucins produced by these cells display glycosylation patterns associated with tumors, which may not represent patterns observed in the steady state intestine. Thus, to better study steady-state intestinal mucin effects on DCs and given the high percentage of homology between mouse and human mucins (12, 13), we repeated experiments using mucin purified from mouse intestine. We purified mucin from either small or large intestine, with MS analysis showing a similar composition between the two preparations, with Muc2 being the predominant component (Tables S2 and S3).

To determine whether mouse intestinal mucins were able to induce IL-8 expression, human moDCs were stimulated with increasing concentrations of large intestinal mucin. At both the RNA (Fig. 1*D*) and protein level (Fig. 1*E*), IL-8 was significantly up-regulated by human moDC in response to murine large intestinal mucin. A dose-dependent enhancement was observed, with a dose of 50 μg/ml inducing a significant increase in IL-8 expression (Fig. 1, *D* and *E*).

Next, the capacity of large intestinal mucins to promote IL-8 expression was compared with small intestinal mucins, to determine any location-specific properties of the mucins. Significant up-regulation of IL-8 expression was observed with both small and large intestinal mucins (Fig. 1*F*), suggesting that this property is apparent for mucin obtained from different intestinal locations and not restricted to the large intestine. Although a higher induction of IL-8 was observed in the presence of large intestinal mucins *versus* small intestinal, this difference was not significant. Together, these data suggest that intestinal mucins isolated from healthy mice show similar properties to human colonic cell-derived mucins, with both causing up-regulation of the pro-inflammatory chemokine IL-8 by human DC.

**Figure 2.**
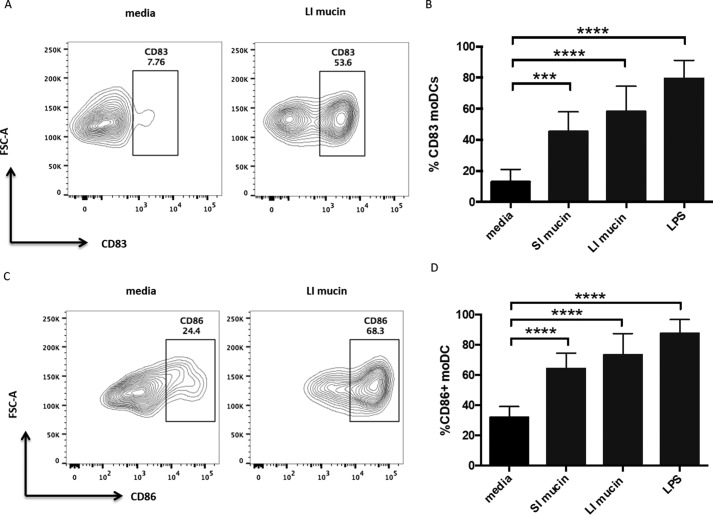
**Mouse intestinal mucin can induce up-regulation of DC activation markers.** Human moDC were treated with mucin (50 μg/ml) or LPS (10 ng/ml), and activation markers were detected by flow cytometry. *A* and *C*, representative flow cytometry plots showing moDC expression of CD83 (*A*) and CD86 (*C*) either untreated or treated with large intestinal (LI) mucin. *B* and *D*, percentages of CD83^+^ moDCs (*B*) and CD86^+^ moDCs (*D*) untreated and treated with small intestinal mucin (*SI*), large intestinal mucin (*LI*), and LPS. *n* = 8 independent experiments. ***, *p* < 0.001; ****, *p* < 0.0001 assessed via one-way ANOVA followed by Dunnet's multiple comparison test.

It is possible that enhanced expression of IL-8 by DCs was linked to the ability of the mucins to activate the DCs. To determine whether treatment of DCs with mucins resulted in DC activation, expression of CD86 and CD83, classical DC activation markers, was measured by flow cytometry in the presence and absence of mucin. As expected, both DC activation markers were highly induced by the TLR4 ligand lipopolysaccharide (LPS) (Fig. 2). When treated with either small intestinal or large intestinal mucin, moDCs also showed significantly enhanced expression of activation markers, with increased populations of both CD83^+^ (Fig. 2, *A* and *B*) and CD86^+^ DC (Fig. 2, *C* and *D*). Thus, consistent with the induction of the pro-inflammatory chemokine IL-8, intestinal mucins promote pro-inflammatory features on moDCs.

### Mouse intestinal mucins induce DC activation in a TLR4- and bacterial DNA-independent manner

**Figure 3.**
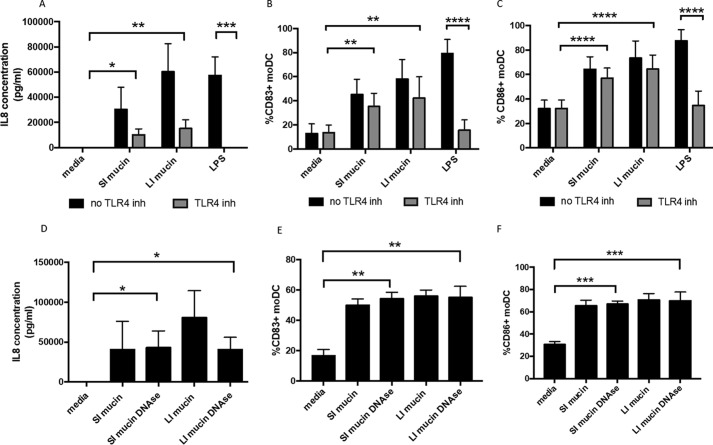
**Mucin-induced DC activation and IL-8 production are independent of LPS and DNA.** moDCs were treated with small intestinal (*SI*) and large intestinal (*LI*) mucins (50 μg/ml) in the absence (*black bars*) and the presence (*gray bars*) of TLR4 inhibitor CLI-095. LPS (10 ng/ml) was used as a control. *A*, ELISA was performed to detect IL-8 in supernatants from untreated and mucin-treated moDCs. *n* = 8 independent experiments. *, *p* < 0.05; **, *p* < 0.01; ***, *p* < 0.001 assessed using Kruskal–Wallis test followed by Dunn's multiple comparison test. *B* and *C*, percentages of CD83^+^ (*B*) and CD86^+^ (*C*) moDCs either untreated or treated with mucin (SI and LI) or LPS, in the presence of the TLR4 inhibitor. *n* = 8 independent experiments. **, *p* < 0.01; ****, *p* < 0.0001 assessed using one-way ANOVA followed by Dunnet's multiple comparison test. *D*, detection of IL-8 in supernatants from moDCs either left untreated or treated with mucin or DNase-treated mucin (SI and LI, 50 μg/ml) by ELISA. *n* = 4 independent experiments. *, *p* < 0.05 assessed using Kruskal–Wallis test followed by Dunn's multiple comparison test. *E* and *F*, percentages of CD83^+^ (*E*) and CD86^+^ (*F*) moDCs either left untreated or treated with mucin or DNase-treated mucin (SI and LI). *n* = 4 independent experiments. **, *p* < 0.01; ***, *p* < 0.001 assessed using one-way ANOVA followed by Dunnet's multiple comparison test.

Given that bacterial endotoxin LPS is a potent inducer of IL-8 expression and DC activation (14, 15), and mucins purified from murine intestine could conceivably co-purify with bacterial products from the microbiota, one explanation for the results above was that DC stimulation by mucins was due to the presence of LPS in mucin samples. Although we did detect some levels of LPS in mucin samples from the large intestine (0.96 ng/ml), much lower levels were detected in our murine small intestinal mucins (0.075 ng/ml), consistent with the higher microbiota levels in the large *versus* small intestine. However, despite these differences, there was no appreciable difference in IL-8 (Fig. 1*D*) or DC activation markers (Fig. 2, *A–D*) induced by small *versus* large intestinal mucin. However, to directly test whether LPS contamination of intestinal mucin samples was responsible for their induction of DC activation and IL-8 production, we used a TLR4 inhibitor (CLI-095), which binds to the intracellular domain of TLR4, preventing downstream pro-inflammatory effects (16, 17). The TLR4 inhibitor completely prevented LPS-induced IL-8 production (Fig. 3*A*) and DC activation (Fig. 3, *B* and *C*) by human DCs, inhibiting the effects of 10 ng/ml LPS (which is >10× higher than LPS levels in diluted mucin samples). Although the TLR4 inhibitor caused a reduction in IL-8 levels induced in the presence of mucins, both small and large intestinal mucins still significantly induced IL-8 expression by moDC when TLR4 signaling was blocked (Fig. 3*A*). These results were also confirmed by cytometric bead array (CBA; Fig. S2*A*). However, there was no TLR4-independent induction of other pro-inflammatory cytokines tested (TNFα, IL-6, and IL-23) or the anti-inflammatory cytokine IL-10 (Fig. S2, *B–E*).

Additionally, both small and large intestinal mucin preparations significantly induced both DC activation markers, CD83 (Fig. 3*B*) and CD86 (Fig. 3*C*), in the presence of the TLR4 inhibitor, with no significant difference observed between untreated and TLR4 inhibitor-treated cells. These results suggest that although contaminating LPS may cause some induction of IL-8, murine intestinal mucin significantly enhances DC activation and IL-8 production in a TLR4-independent manner.

Additionally, it was also possible that bacterial DNA, containing CpG motifs that can activate DCs via TLR9, could co-purify with mucin samples to cause DC activation. Indeed, DNA was detected in purified mucin samples by gel electrophoresis (Fig. S3). However, when samples were treated with DNase, which eliminated DNA from samples (Fig. S3), both small and large intestinal mucin still induced significant levels of IL-8 expression by human DC, with no significant difference observed *versus* untreated mucin (Fig. 3*D*). Similar results were observed for induction of DC activation (Fig. 3, *E* and *F*). Therefore, these results suggest that DC activation and IL-8 induction by intestinal mucins is not a result of any bacterial or other DNA contamination in the mucin preparations.

### DNA-free mucin glycopeptides induce IL-8 production and DC activation

**Figure 4.**
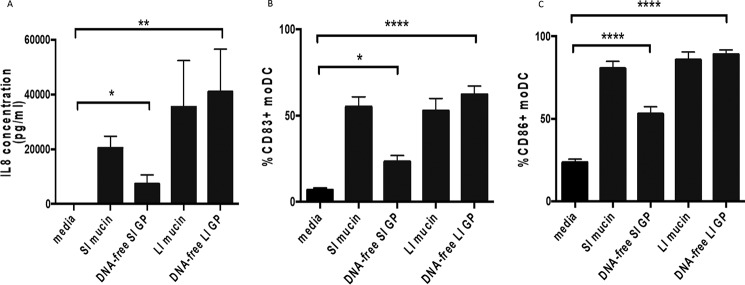
**DNA-free mucin glycopeptides induce IL-8 production and DC activation.** moDCs were treated with small intestinal (*SI*) and large intestinal (*LI*) mucins (50 μg/ml) and mucin glycopeptides (50 μg/ml, previously treated with DNase). *A*, detection of IL-8 in supernatants from moDCs either left untreated, treated with intact mucin (SI and LI), or treated with mucin glycopeptides (*DNA-free SI GP* and *LI GP*) by ELISA. *n* = 6 independent experiments. *, *p* < 0.05; **, *p* < 0.01 assessed using Kruskal–Wallis test followed by Dunn's multiple comparison test. *B* and *C*, percentages of CD83^+^ (*B*) and CD86^+^ (*C*) moDCs either untreated or treated with mucin (SI and LI) or mucin glycopeptides (DNA-free SI GP and LI GP). *n* = 6 independent experiments. *, *p* < 0.05; ****, *p* < 0.0001 assessed using Kruskal–Wallis test followed by Dunn's multiple comparison test.

To elucidate whether protein-rich or sugar-rich mucin regions of Muc2 could be responsible for the induction of DC activation and IL-8 production, we generated mucin glycopeptides from DNA-free mucin by trypsin digestion, which digest nonglycosylated protein-rich parts of Muc2. Additionally, trypsin digestion may eliminate other contaminating protein(s) that co-purified with mucins. Thus, mucin samples were reduced, alkylated, and then treated with trypsin to digest the mucin and other proteins. Generation of mucin glycopeptides was confirmed by SEC–MALLS, because the molecular mass is reduced compared with the unreduced mucin (0.7–1.6 *versus* 4–6 MDa) (Table S4). Both DNA-free small and large intestinal mucin glycopeptides significantly induced IL-8 expression (Fig. 4*A*). Although small intestinal mucin glycopeptides did have a reduced ability to induce IL-8 expression *versus* undigested small intestinal mucin samples (Fig. 4*A*), no significant differences were observed comparing untreated large intestinal mucin and large intestinal mucin glycopeptides (Fig. 4*A*). Similar results were observed in DC activation (Fig. 4, *B* and *C*). Additionally, IL-8 production and DC activation were still significantly increased in the presence of a TLR4 inhibitor (Fig. S4). Together, these results indicate that mucin-induced IL-8 production in human DCs is not inhibited by the digestion of protein-rich mucin regions and suggest that mucin glycans may be involved in the modulation of DC function.

### Glycans contribute to induction of IL-8 in mucin-treated DCs

Next, potential mechanisms by which mucins can induce DC activation and IL-8 production were analyzed. Because 80% of the mass of mucins is due to glycan side chains, we tested the importance of glycosylation on mucin-induced DC modulation, by treating mucins to modify or remove terminal sugars on the glycans. First, mouse large intestinal mucins were treated with sodium metaperiodate (NaIO_4_), which oxidizes vicinal diols, cleaving them and producing two aldehydes and modifying terminal mucin glycans (18). Mucin oxidation was verified by detection of samples with Schiff's reagent staining via slot blotting (Fig. S5).

**Figure 5.**
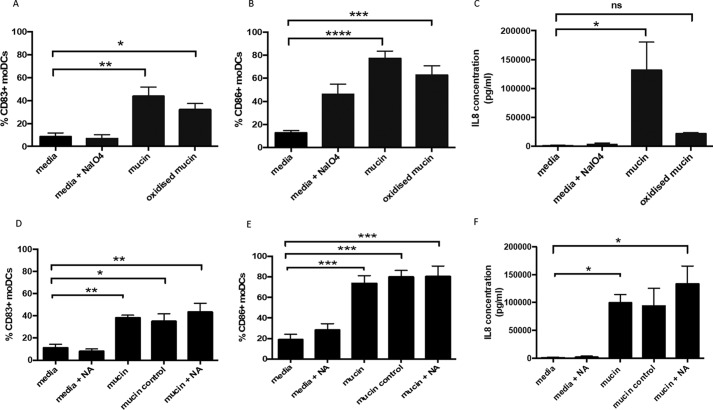
**Mucin-induced IL-8 production is glycosylation-dependent but sialic acid–independent.** moDCs were treated with media containing sodium metaperiodate (NaIO_4_), large intestinal mucin (50 μg/ml), or oxidized mucin (treated with NaIO_4_) (50 μg/ml). *A* and *B*, percentages of CD83^+^ (*A*) and CD86^+^ (*B*) moDCs determined by flow cytometry. *C*, levels of IL-8 in supernatant determined by ELISA. The results are from four independent experiments, and statistical significance was assessed in *A* and *B* using one-way ANOVA followed by Dunnet's multiple comparison test (*, *p* < 0.05; **, *p* < 0.01; ***, *p* < 0.001; ****, *p* < 0.0001) and assessed in *C* using the Kruskal–Wallis test followed by Dunn's multiple comparison test (*, *p* < 0.05). Neuraminidase-treated (*NA*) mucin (50 μg/ml) was cultured with moDC. moDCs were also treated only with neuraminidase, intact mucin (50 μg/ml), and mucin control (untreated with neuraminidase but incubated under digestion conditions). *D* and *E*, percentage of CD83^+^ (*D*) and CD86^+^ (*E*) moDCs determined by flow cytometry. *F*, IL-8 levels determined by ELISA. The results are from four independent experiments, and statistical significance was assessed in *D* and *E* using one-way ANOVA followed by Dunnet's multiple comparison test (*, *p* < 0.05; **, *p* < 0.01; ***, *p* < 0.001) and assessed in *F* using the Kruskal–Wallis test followed by Dunn's multiple comparison test (*, *p* < 0.05).

Oxidized mucin causes an enhancement of CD83 expression on DC, which is statistically significant compared with non–mucin-treated DCs but not to intact mucin-treated DCs (Fig. 5*A*). Similarly, CD86 expression by DC is significantly enhanced by oxidized mucin treatment, but it is not significantly different compared with intact mucin-treated DCs (Fig. 5*B*), suggesting that up-regulation of co-stimulatory marker by mucins is not dependent on the glycan oxidation state. However, surprisingly, oxidized mucin did not significantly enhance production of IL-8 by DC (Fig. 5*C*). Thus, mucin glycans appear to be important in promoting IL-8 induction by DC, but not in regulation of co-stimulatory molecules.

Next, to further study the contribution of mucin glycosylation in modulation of DC function, neuraminidase-mediated removal of sialic acid was performed, with effective removal confirmed by a large reduction in *Sambucus nigra* lectin and *Maackia amurensis* lectin II (MAL II) staining of the mucin after slot blotting, indicating reduced presence of α2-3– and α2-6–linked sialic acid (Fig. S6). Neuraminidase-treated mucin still induced a significant increase in CD83 and CD86 expression by DCs, akin to nontreated mucin (Fig. 5, *D* and *E*). However, in contrast to reduced IL-8 induction by oxidized mucin, neuraminidase-treated mucin still induced significantly increased IL-8 production by moDCs (Fig. 5*F*). Together, these results suggest that sialic acid is not involved in the induction of IL-8 secretion by DCs or induction of co-stimulatory molecules.

### Treatment of moDCs with mucin induces recruitment of neutrophils in an IL-8–dependent manner

We next determined whether changes to DCs after interaction with mucins resulted in any functional consequences. Because DC production of IL-8 is induced upon interaction with mucins and IL-8 is an important pro-inflammatory chemokine that recruits immune cells expressing the chemokine receptor CXCR1 such as neutrophils (11), we investigated whether DC treatment with mucins altered the ability to attract neutrophils via a transmigration assay.

**Figure 6.**
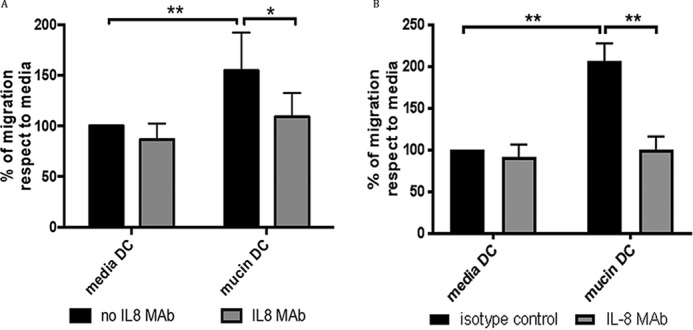
**Neutrophil-like cells are recruited by mucin-induced IL-8 from moDCs.** Transmigration of the neutrophil cell line HL60 was evaluated in transmigration assays across endothelial cell monolayers (EaHY926 cells). *A*, HL60 cells were placed into the upper well of a transmigration well, with supernatant from untreated moDC or moDC treated with 10 μg/ml mucin for 24 h. Transmigration of HL60 cells toward untreated DC or mucin-treated DC supernatants was carried out in the absence and the presence of anti–IL-8 blocking antibody. Migration is expressed as a percentage of migrated cells calculated compared with the transmigration observed in the presence of supernatants from untreated moDCs. *n* = 4 independent experiments. *, *p* < 0.05; **, *p* < 0.01 assessed using one-way ANOVA followed by Dunnet's multiple comparison test. *B*, transmigration of primary human neutrophils was similarly evaluated with supernatant from untreated moDC or moDC treated with 10 μg/ml mucin for 4 h, in the absence and the presence of anti–IL-8 blocking antibody. *n* = 3 independent experiments. **, *p* < 0.01 assessed using one-way ANOVA followed by Dunnet's multiple comparison test.

First, using the neutrophil cell line HL60 (19–21), we tested their ability to transmigrate across an endothelial cell monolayer, toward supernatants from untreated DCs or mucin-treated DCs. Significantly increased transmigration of HL60 cells was observed in the presence of supernatant from DCs treated with large intestinal mucin (Fig. 6*A*). This transmigration was IL-8–dependent, because an anti–IL-8–blocking antibody completely inhibited the mucin-induced enhancement of neutrophil migration (Fig. 6*A*). Similarly, a significant increase in neutrophil transmigration was observed in the presence of supernatants from moDCs treated with mucin in the presence of TLR4 inhibitor (Fig. S7*A*).

We also tested the ability of primary neutrophils isolated from human blood to transmigrate in the presence of mucin-treated moDC supernatant. Similar to the HL60 cells, primary neutrophils showed a significantly increased migration toward supernatant from mucin-treated moDC, which was reversed in the presence of an anti–IL-8 antibody (Fig. 6*B*). Again, use of a TLR4 inhibitor did not reduce the ability of primary neutrophils to migrate toward mucin-treated moDC supernatant (Fig. S7*B*). Thus, these data suggest that interaction of mucins with DCs can promote the ability of these cells to enhance neutrophil migration via production of IL-8.

## Discussion

The intestinal mucus layer, composed predominantly of the mucin MUC2/Muc2, plays an important role in intestinal barrier protection. Recent work suggests that, in addition to being a physical barrier, the intestinal mucin layer may also have active immunomodulatory functions via interaction with DCs (22). Although original studies suggest that Muc2 promotes anti-inflammatory responses from DCs, our work provides a new point of view for the role of the intestinal mucus barrier. Our data suggest that DCs might recognize mucins as danger signals, promoting DC activation and IL-8 production that can promote neutrophil migration. Such DC activation may occur during disruption of epithelial barrier integrity, facilitating the contact between mucin and intestinal DCs and triggering inflammatory responses against invading pathogens.

Similar to previous studies (8, 22, 23), we studied interactions between purified mucins and human monocyte-derived DC, given feasibility issues in obtaining enough human intestinal DCs for the studies performed. These cells were differentiated in similar conditions to those used by Shan *et al.* (22). However, we were not able to replicate results from these studies (8, 22, 23). Ishida *et al.* (8) reported induction of apoptosis and reduced expression of CD83 on moDC via MUC2 treatment. However, we did not observe reduced cell viability and, in contrast, observed increased CD83 expression on mucin-treated moDCs (24). Similarly, although Ohta *et al.* (23) reported that MUC2 could prevent LPS-induced IL-12 expression on moDCs, we did not observe any ability of mucin to prevent expression of pro-inflammatory molecules in the presence of LPS (Fig. S8). Shan *et al.* (22) reported that both MUC2 from LS174T cells and mouse small intestinal mucin prevented inflammatory DC responses such as increased expression of DC activation markers and production of pro-inflammatory mediators. Additionally, enhanced production of the anti-inflammatory cytokines IL-10 and transforming growth factor-β and increased RALDH activity were reported in the presence of mucin and LPS in human myeloid CD1c^+^ DCs (24). In our results, using similar mucin concentrations, we have shown that both enriched human MUC2 preparations and mouse intestinal mucins from both small and large intestine can promote pro-inflammatory features in monocyte-derived DCs such as IL-8 production and DC activation. Even in the presence of LPS, our mucin samples promoted additional IL-8 production by moDCs (Fig. S8), and we did not find increased IL-10 production on moDCs in our assays (Fig. S2*B*). We also analyzed the expression of other relevant pro-inflammatory human cytokines including IL-6, IL-23, and TNFα by CBA, but they were not significantly increased in a TLR4-independent manner (Fig. S2, *C–E*).

The reason for the discrepancies between studies is not readily apparent; however, different protocols were employed to isolate Muc2/MUC2. Here we collected GuHCl-soluble mucin (in a single extract) and purified the mucin by denaturing CsCl density gradient centrifugation. In contrast, Shan *et al.* (22) performed repeated GuHCl extractions; each was followed by high-speed centrifugation and finally isolated an insoluble Muc2 residue via reduction of disulfide bonds. Detailed comparison is not possible because the properties and composition of the mucin preparation employed in the Shan study are not reported. The Muc2 employed by us most likely contains more easily solubilized Muc2. Whether the two protocols result in Muc2 preparations with different properties, in particular enriching in mucins with differing glycosylation, is not known. Alternatively, we cannot rule out that differences in the mouse microbiota between laboratories may affect the glycosylation displayed by mucins, either influencing the biosynthetic machinery adding the glycans and/or via the action of microbial glycosidases. Thus, further work is required to determine how different mucin preparations can alter DC function.

As we have shown, our experiments suggest that mucins may be able to induce IL-8 expression on human moDCs. IL-8 is a potent chemotactic factor expressed by different immune cells, including monocytes and macrophages, which can recruit cells expressing the chemokine receptors CXCR1 and CXCR2 such as neutrophils, T cells, and monocytes/macrophages. IL-8 is expressed at low levels during steady-state conditions but can be highly induced by pro-inflammatory agents, such as TNFα, IL-1, interferon-γ, and LPS, and stress factors such as reactive oxygen species (25). TLR stimulation via the adaptor protein MyD88 can activate NF-κβ and MAPK signaling pathways, which also can induce IL-8 expression. Because different bacterial PAMPs can bind to TLRs including LPS to TLR4 and CpG-containing DNA can bind to TLR9 (26), a main concern for our studies was that bacterial contaminants present in mucin preparations induced IL-8 production and DC activation. We performed our experiments in the presence of a TLR4 inhibitor to rule out the possibility that contaminant LPS was inducing IL-8 or DC activation. Additionally, we removed the DNA present in the sample, which may come either from epithelial cells or bacteria, and this did not affect IL-8 production or DC activation. We cannot exclude the possibility that other bacterial by-products may induce the effects observed. However, our human mucin preparation, which does not contain bacterial products, also induced IL-8 expression, and thus we do not think this is the case.

Importantly, we have shown here that mucin glycans may be involved in the induction of IL-8 and DC activation. First, we treated our DNA-free mucin preparations with trypsin to degrade bacterial proteins and mucin protein regions that are not heavily glycosylated (N-terminal, C-terminal, and internal CysD domains). Our results suggest that mucin glycopeptides can still induce IL-8 production and DC activation. However, small intestinal mucin glycopeptides showed a lower ability compared with large intestinal glycopeptides to induce these effects, which may be attributed to the loss of either contaminant proteins or lowly glycosylated mucin regions. Both small and large intestinal glycopeptides were able to induce IL-8 and DC activation in the presence of the TLR4 inhibitor (Fig. S4), which again suggests that effects seen are TLR4-independent. Interestingly, despite the ability of intestinal mucin to induce IL-8 and co-stimulatory molecules, there appears to be no effect on production of other pro-inflammatory cytokines (TNFα, IL-6, and IL-23) or the anti-inflammatory cytokine IL-10 (Fig. S2). We cannot rule out that mucin may affect other pro- or anti-inflammatory cytokine production by DCs, but our data does at least point to a robust induction of IL-8. The reasons for the up-regulation of IL-8 and co-stimulatory molecules, but not other pro-inflammatory cytokines tested here, remain unclear but likely stem from the triggering of specific signaling pathways by intestinal mucin that induce some aspects of DC activation but not others. Indeed, it is well established that different stimuli can cause differential induction of activation markers to induce different types of T-cell response (27). Although our data suggest that mucin-mediated effects on DCs do not occur through TLR4 signaling or via bacterial DNA, it will be interesting to determine whether mucin-mediated effects occur via other classical pro-inflammatory pathways (*e.g.* MyD88- or TRIF-mediated triggering of NF-κB and MAP kinase signaling) or whether novel pathways are responsible. Thus, further work is required to determine how mucins trigger signaling in DCs to promote a selective pro-inflammatory phenotype, which is outside the purview of this current study.

We also treated mucin preparations with sodium metaperiodate, which effectively oxidizes glycans on the mucin, as shown by Schiff's staining. Oxidized mucin induced some IL-8 induction but was not significant compared with the untreated moDCs. We did not observe a significant difference comparing treatments with oxidized and native mucin, although there is a trend suggesting a decreased pro-inflammatory effect. Interestingly, there was no alteration in the ability of oxidized mucin to induce the DC activation markers CD83 and CD86, suggesting that different mechanisms are responsible for mucin-mediated induction of IL-8 production and DC maturation. However, more assays are required to confirm the idea that our oxidized mucin lost its pro-inflammatory ability, and thus glycans are essential in the induction of IL-8 production and to determine mechanisms responsible for induction of DC activation markers. The use of sodium metaperiodate to validate the effect of mucin glycans has also been reported by Cobo *et al.* (28), who showed that treatment of MUC2 with sodium metaperiodate–abrogated mucin-induced β-defensin 2 production by epithelial cells. The authors also tested whether digestion with neuraminidase and mannosidase affected β-defensin 2 production, but did not observe significant differences compared with native mucin. Similarly, we did not observe a reduced ability of mucins to induce IL-8 secretion in the presence of neuraminidase-treated mucin. These findings suggest that the pro-inflammatory effects that we observe are not due to sialic acid moieties present on mucins. In concordance, a free synthetic sialic acid did not result in significant induction of IL-8 production or DC (Fig. S9). Additionally, sialic acid did not reduce LPS-mediated IL-8 production or DC activation, as previously shown in the literature (23), which suggests that this glycan does not have immunomodulatory effects on DC. Thus, other glycans apart from free sialic acid may be involved in mucin-induced effects on moDC. However, given that sialic acid levels were reduced but not eliminated by neuraminidase treatment (Fig. S6), we cannot rule out that the remaining residual sialic acid levels are contributing to the effects we observe on DCs. Thus, further work is required to determine the exact glycan moieties responsible for effects on DC. Aberrant glycosylation of mucins has been reported in patients with inflammatory bowel disease and colorectal cancer, and this may contribute to disease pathogenesis (29, 30). Because glycosylation of mucins appears to play a key role in inducing pro-inflammatory responses in human moDCs, identification of the functionally important glycans might provide novel clinical targets, with blocking agents, such as specific lectins potentially of clinical benefit. Additionally, such work will be important to elucidate the signaling pathways involved in these effects, which may also represent important clinical targets.

Moreover, we explored whether moDC-secreted IL-8 was able to play any functional roles. Because IL-8 is an important chemoattractant, we tested the ability of moDCs supernatants to recruit neutrophils, which express the chemokine receptor CXCR1. To test this possibility, we used the well characterized cell line HL-60, which can be differentiated to neutrophil-like cells, as well as primary human blood neutrophils. We observed that supernatants from mucin-treated moDCs were able to recruit neutrophils in an IL-8–dependent manner. Because LPS may be partially responsible for inducing IL-8 and thus transmigration in our assays, we also used supernatants from moDC treated with mucin in the presence of the TLR4 inhibitor. These supernatants also induced significant neutrophil transmigration in our assays (Fig. S7), which suggests that mucin-induced IL-8 may induce neutrophil recruitment. Because neutrophils are important mediators of early inflammation in the intestine and are rapidly increased during inflammation, mucins might play a role recruiting neutrophils to the intestinal mucosa, when barrier integrity is compromised and invaded by enteric pathogens. Taken together, our results suggest that DCs may recognize mucins as danger signals, which induce up-regulation of DC activation markers and secretion of IL-8, which could recruit pro-inflammatory mediators to the intestine.

Here, we have shown that MUC2/Muc2 has the potential to induce pro-inflammatory responses, inducing IL-8 expression and DC activation in human moDCs, which may be important in the context of different inflammatory conditions such as Crohn's disease and ulcerative colitis. Thus, understanding the interactions between DCs and mucin may provide interesting molecular targets that could prevent exacerbated inflammation.

## Experimental procedures

### Mucin isolation

The human colon adenocarcinoma cell line LS174T (European Collection of Cell Culture) was used as a source of glycosylated human intestinal mucin, including mucin MUC2 (10). This cell line was cultured using Dulbecco's modified Eagle's medium (DMEM) supplemented with 10% (v/v) FCS, 1% l-glutamine, and 1% penicillin/streptomycin (all from Invitrogen, UK), and supernatants used for mucin purification. WT C57BL/6 mice were maintained under specific pathogen-free conditions in the Biological Unit Services Unit at the University of Manchester and used at 6–12 weeks old. All procedures were performed in accordance with the Home Office Scientific Procedures Act (1986) and under the DERFA license. Small or large intestine was excised, cut longitudinally, and washed with PBS to remove feces. Mucus was scraped into 6 m GuHCl (Sigma–Aldrich), solubilized by rotating at 4 °C for 24–48 h, and then centrifuged for 10 min at 3000 × *g* to remove insoluble material.

### Purification of mucins by isopycnic density gradient centrifugation

Isopycnic density gradient centrifugation was performed to purify mucins from LS174T cell culture supernatants or mouse intestinal mucus as described (31). Briefly, cell culture supernatants or mouse intestinal mucus samples were adjusted to 4 m GuHCl and subjected to CsCl with 4 m GuHCl density-gradient ultracentrifugation at a starting density of 1.4 g/ml at 100,000 × *g* for 66 h at 15 °C (Beckman 70 Ti rotor). After centrifugation tubes were emptied from the top into 20 fractions, and mucins were detected by immunostaining with a Muc2-specific antiserum (32, 33) or by periodic acid–Schiff staining (34). Mucin-rich fractions were pooled and subjected to a CsCl with 0.2 m GuHCl density-gradient centrifugation at a starting density of 1.5 g/ml (100,000 × *g* for 66 h at 15 °C). Mucin-rich fractions were finally pooled and dialyzed in 1× PBS and concentrated using a 10-kDa molecular mass cutoff Vivaspin centrifugal concentrator.

Density was estimated by weighing 1 ml of every fraction, and absorbance at 280 nm was measured using a S-22 UV-visible spectrophotometer (Boeco) to determine fractions enriched in other proteins and/or nucleic acids. Additionally, to detect nucleic acids, samples were loaded in 0.7% agarose gels and stained with SafeView dye (NBS-SV1).

### SEC–MALLS of mucin preparations

To determine the molecular mass distribution and concentration of mucin preparations, samples were analyzed by SEC–MALLS after chromatography on Shodex columns connected in series (OHpak SB-806M HQ) (for intact mucins) or a Superose 6 10/300 (for reduced mucin and glycopeptides) column, eluted with 0.2 m sodium chloride, 1 mm EDTA, and 0.05% sodium azide. Column eluents were passed through an inline DAWN EOS laser photometer and an Optilab rEX refractometer with QELS dynamic light scattering attachment. Analysis was performed using ASTRA version 6 software.

### Analysis of mucin preparations by tandem MS

Following trypsin digestion of mucins, peptides were concentrated and desalted using solid-phase extraction with ZipTips (Millipore). The samples were analyzed by LC–MS/MS using an UltiMate 3000 rapid separation LC coupled to a LTQ Velos Pro mass spectrometer. To analyze MS data, the in-house MASCOT (Matrix Science) search engine was used, and fragmentation data were searched against the Uniprot database (35). MASCOT search data were analyzed using Scaffold 3.0 (Proteome software), and a number of exclusive unique peptides was obtained, with a peptide probability of >95%.

### DNase digestion

Mucin was treated with 10 units/ml DNase I (grade II; Roche Applied Science), 5 mm MgCl_2_ (Promega), and 50 mm Tris-HCl (United Biochemicals) and incubated for 1 h at 37 °C, and the reaction stopped by adding 25 mm EDTA and heating for 10 min at 75 °C. To remove digestion products and exchange the buffer, the samples were spun in 10-kDa molecular mass cutoff Vivaspin filters, replacing the digestion media for 1× PBS. The degree of DNA digestion was checked by running the sample in a 0.7% agarose gel, stained with SafeView dye. Mucin concentration was determined by SEC–MALLS.

### Generation of DNA-free mucin glycopeptides

DNA-free mucin was reduced with 10 mm DTT for 3 h and alkylated with 25 mm iodoacetamide, protected from light, for 45 min at room temperature. Buffer was exchanged in a 10-kDa molecular mass cutoff Vivaspin filters, replacing the solution for 0.1 m ammonium bicarbonate. Subsequently, proteomic grade trypsin (Promega) was added in a final protease:protein ratio of 1:10, as recommended by the manufacturer, and incubated overnight at 37 °C. To ensure complete digestion, fresh trypsin was added the following day and incubated for 3 h. After digestion, samples were spun in 10-kDa molecular mass cutoff Vivaspin filters, replacing the digestion media for 1× PBS.

### Sodium metaperiodate

Mucins were treated with 3 mm sodium metaperiodate (Thermo Scientific) on ice for 1 h, diluting the sodium metaperiodate and mucin in a ratio 1:1. To remove sodium metaperiodate and exchange the buffer, the samples were spun in 10-kDa molecular mass cutoff Vivaspin filters, replacing the sodium metaperiodate for 1× PBS. Oxidation was verified by slot blotting followed by periodic acid–Schiff staining, omitting the first oxidation step with periodic acid. The buffer was exchanged with 1× PBS using 10-kDa molecular mass cutoff Vivaspin filters.

### Neuraminidase treatment

Mucins were treated overnight with different concentrations of neuraminidase (New England BioLabs) including 5× Sigma reaction buffer or 10× New England BioLabs reaction buffer, at 37 °C. The reaction was stopped by heating the sample at 100 °C for 5 min. To confirm that neuraminidase digestion was effective, *S. nigra* lectin or *M. amurensis* lectin II (Vector Labs) was used to detect for the presence of α2-3– or α2-6–linked sialic acid on mucins (33).

### Endotoxin analysis

Endotoxin concentration was measured using a limulus amebocyte lysate chromogenic endotoxin quantitation kit (Thermo Scientific), following the manufacturer's protocol.

### Generation of monocyte-derived DC

Buffy coat or apheresis cones from healthy donors, provided by the National Health Service blood bank (Manchester, UK), and peripheral blood mononuclear cells (PBMCs) obtained from using Ficoll–Paque Plus (GE Healthcare) according to the manufacturers' protocols. CD14^+^ monocytes were isolated from PBMCs using anti-human CD14 antibody-coated beads (Miltenyi Biotech, Surrey, UK) and incubated in RPMI 1640 medium containing 10% FCS, 1% penicillin/streptomycin, and 1% l-glutamine, at a concentration of 2 million cells/ml, with 50 ng/ml IL-4 and 50 ng/ml granulocyte/macrophage colony-stimulating factor (PeproTech, London, UK) for 6–7 days. Medium and cytokines were refreshed after 3 days, and the cells were plated into fresh wells on day 6 at a concentration of 1 million cells/ml. In some experiments, the cells were then treated with 10 ng/ml LPS (Sigma–Aldrich), mouse small or large intestinal mucin (50 μg/ml), or human MUC2 (50 μg/ml) isolated from LS174T cells for 22–24 h before analysis by flow cytometry. In some experiments, the cells were preincubated with 1 μg/ml of the TLR4 inhibitor CLI-095 (InVivoGen) for 1 h at 37 °C.

### Flow cytometry

Cells were analyzed by flow cytometry using the following anti-human antibodies: anti-CD14 (clone M5E2; BioLegend, London, UK), anti-CD86 (clone IT2.2; BioLegend), anti-CD83 (clone HB15e, BD Bioscience, Oxford, UK), anti-CD11c (clone 3.9; BioLegend), anti-CD1c (clone L161; BioLegend), anti-HLA-DR (clone, L243; BioLegend), anti-CD13 (clone WM15; BioLegend), anti-CD33 (clone P67.6; BioLegend), anti-CD141 (clone 1A4; BD Bioscience, Oxford, UK), and anti-CD11b (clone ICRF44; eBioscience). Dead cells were identified using fixable Viability dye Zombie UV (BioLegend, UK). The data were acquired on a LSRII flow cytometer or Fortessa (BD Bioscience) and analyzed using FACSDiva (BD Bioscience) or FlowJo software (Tree Star, Ashland, Oregon).

### Real-time qPCR

Total RNA was purified using the RNeasy mini kit (Qiagen), following the manufacturer's instructions. cDNA was prepared using the GoScript reverse transcription system (Promega). Real-time qPCR was performed using an AB Biosystems real-time PCR system (Life Technologies), using Fast SYBR green master mix (AB Biosystems). The values of target mRNA were corrected relative to the housekeeping gene coding for human β-microglobulin. The samples were incubated for 95 °C for 5min, followed by denaturation for 5 s at 95 °C and combined annealing/extension for 30 s at 60 °C for a total of 40 cycles. The data were analyzed using the 2-ΔΔCT method and expressed as fold changes. Primer sequences used were: IL-8 forward: 5′-TCCTGATTTCTGCAGCTCTGTG-3′; IL-8 reverse, 5′-TGGTCCACTCTCAATCACTCTC-3′; β2-microglobulin forward,5′-CTCCGTGGCCTTAGCTGTG-3′; and β2-microglobulin reverse, 5′-TTTGGAGTACGCTGGATAGCC-3′.

### ELISA

IL-8 was quantified from the supernatant of moDCs by the human IL-8 ELISA Ready-SET-Go! (eBioscience) as per the manufacturer's specifications. IL-8 concentration in each sample was calculated based on the standard curve determined for each plate.

### CBA

Levels of the human cytokines IL-1β, IL-6, IL-8, IL-10, IL-12, IL-18, IL-23, and TNFα were quantified in supernatants from moDC using the LEGENDplex human inflammation panel (BioLegend), as per the manufacturer's specifications.

### Transmigration assay

The promyelocytic leukemia cell line HL60 (ATCC; provided by Dr. Caroline Milner, University of Manchester) was cultured using Iscove's modified Dulbecco's medium (Gibco, Life Technologies) supplemented with 20% (v/v) FCS, 2% l-glutamine, and 1% penicillin/streptomycin (Sigma–Aldrich). To differentiate these cells into neutrophil-like cells, 300,000 cells were incubated in culture medium containing with 1.5% (v/v) DMSO for 5 days (19), with differentiation evaluated by measuring CD11b up-regulation by flow cytometry. Ea.hy926 endothelial cells were cultured using DMEM supplemented with 10% (v/v) FCS, 1% l-glutamine, and 1% penicillin/streptomycin (all from Invitrogen). Primary human neutrophils were isolated from peripheral blood by negative selection using the EasySep direct human neutrophil isolation kit (Stemcell Technologies) according to the manufacturer's instructions.

For transmigration assays, 50,000 Ea.hy926 cells in 100 μl of DMEM supplemented with 10% (v/v) FCS, 1% l-glutamine, and 1% penicillin/streptomycin were added onto the top of 6.5-mm Corning Transwell inserts (3.0-μm polyester membrane; Sigma) in a 24-well plate, adding 600 μl of culture medium below the membrane. The cells were incubated overnight at 37 °C and 5% CO_2_, and endothelial monolayer formation was visually confirmed. The inserts were washed with PBS to remove dead and nonadherent cells. Transwells were transferred to fresh 24-well plates containing 600 μl of serum-free X-Vivo 15 medium or serum-free supernatants from moDCs untreated or treated with mucins for 24 h, plus or minus a neutralizing IL-8 mAb (0.4 μg/ml, clone 6217; R&D Systems). A monoclonal mouse IgG1 antibody (0.4 μg/ml, ultra-LEAF, k isotype, clone MG1-45; BioLegend) was used as a control. Differentiated HL60 cells were added to the top of each insert (250,000 cells in 250 μl of X-vivo15 medium) and incubated at 37 °C and 5% CO_2_ for 24 h, and migrated were cells counted. Similarly, primary human neutrophils were added to the top of each insert (250,000 cells in 250 μl of X-vivo15 medium) and incubated at 37 °C and 5% CO_2_ for 4 h, and migrated cells were counted.

### Statistical analysis

Statistical analysis was performed using GraphPad Prism 5 for Mac, version 5.01. All data were expressed as the means plus S.E. For all flow cytometry analysis, the data were compared using repeated measures one-way ANOVA followed by Dunnett's multiple comparison test or unpaired *t* test. For all ELISA experiments, the data were compared using the Kruskal–Wallis test followed by Dunn's multiple comparisons test. A *p* value of < 0.05 was considered significant.

## Author contributions

F. M.-G., A. S. M., D. J. T., and M. A. T. designed the studies and wrote the paper. F. M.-G., T. M. F., C. F., C. S., and A. G. conducted the experiments and analyzed the data.

## Supplementary Material

Supporting Information

## Footnotes

4 The abbreviations used are:

DC: dendritic cell
moDC: monocyte-derived DC
IL: interleukin
LPS: lipopolysaccharide
SEC–MALLS: size-exclusion chromatography–multiangle laser light scattering
qPCR: quantitative PCR
CBA: cytometric bead array
TNF: tumor necrosis factor
DMEM: Dulbecco's modified Eagle's medium
FCS: fetal calf serum
GuHCl: guanidine hydrochloride
PBMC: peripheral blood mononuclear cell
ANOVA: analysis of variance.
