# Supplementary material for: Intestinal mucin activates human dendritic cells and IL-8 production in a glycan-specific manner
Source: J Biol Chem. 2018 Mar 26;293(22):8543–53. doi: 10.1074/jbc.M117.789305 (PMC5986209; doi:10.1074/jbc.M117.789305)
Supplement: Supporting Information [file supp_293_22_8543__index.html]

Intestinal mucin activates human dendritic cells and IL-8 production in a glycan-specific manner — Intestinal mucin activates human dendritic cells and IL-8 production in a glycan-specific manner — Intestinal mucin activates human dendritic cells — Supporting Information 

# Intestinal mucin activates human dendritic cells and IL-8 production in a glycan-specific manner

## Supporting Information

- Supplemental data (.docx, 4.1 MB) - Supplementary data.
