# Supplementary material for: Intestinal mucin activates human dendritic cells and IL-8 production in a glycan-specific manner
Source: J Biol Chem. 2018 Mar 26;293(22):8543–53. doi: 10.1074/jbc.M117.789305 (PMC5986209; doi:10.1074/jbc.M117.789305)
Supplement: Supporting Information [file 10.1074_M117.789305_jbc.M117.789305-1.docx]

**Supplementary material**

Monocyte-derived DC

**CD14**

**HLA-DR**

**CD11c**

**CD1c**


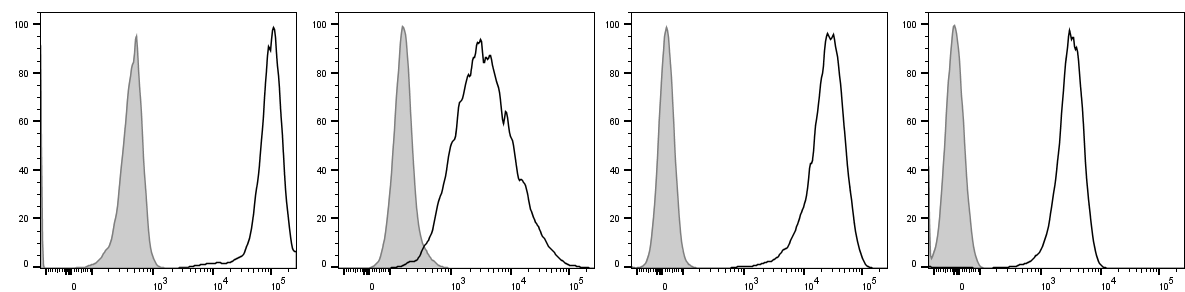

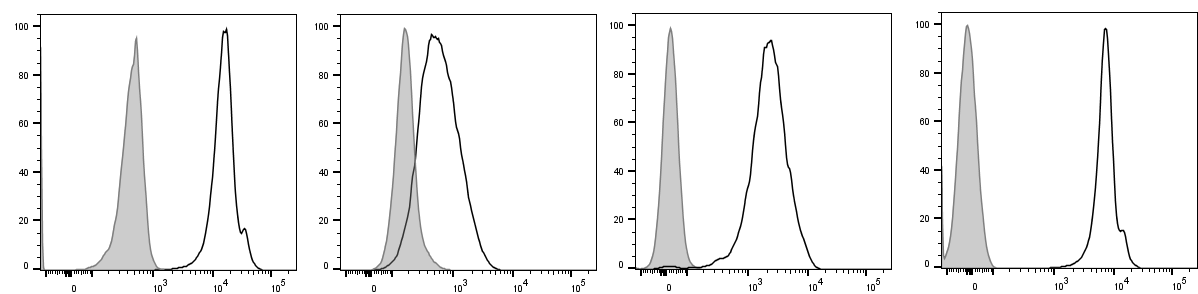


Monocytes

Monocyte-derived DC

**CD11b**

**CD141**

**CD13**

**CD33**

Monocytes


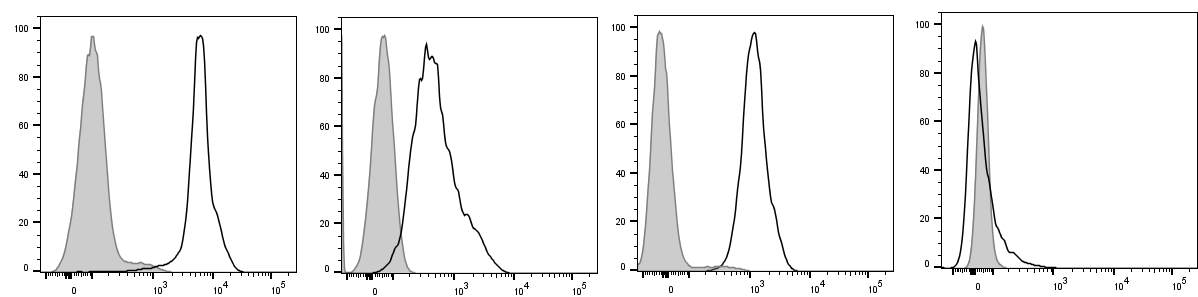

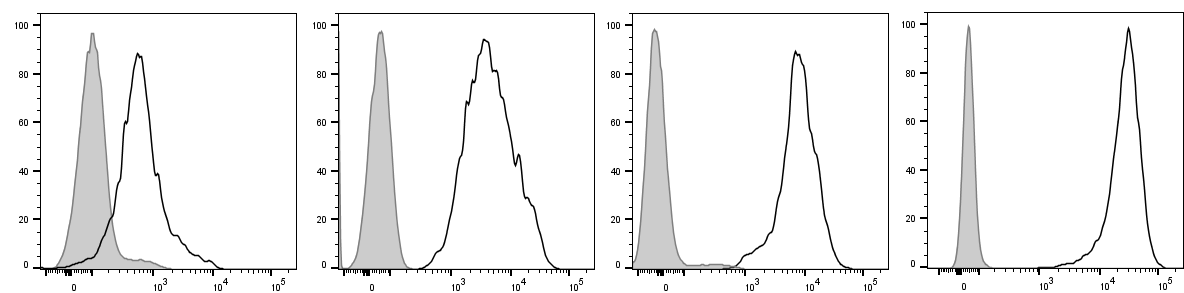


**Figure S1. Purity of human monocyte-derived DC cultures.** Expression of CD14, HLA-DR, CD11c, CD1c, CD11b, CD141, CD13 and CD33 was evaluated on monocytes and cultures of monocyte-derived DCs after 6 days of differentiation by flow cytometry. Representative histograms are shown for each marker (shaded plot, FMO control: non-shaded plot, respective antibody staining).

**A**

**B**

**C**

**D**

**E**

**Figure S2. Intestinal mucins induce IL-8, but not TNF-α, IL-6, IL-23 and IL-10 production by moDCs in a TLR4-independent manner.** moDCs were treated with mouse small intestinal (SI mucin, 50μg/ml) and large intestinal (LI mucin, 50 μg/ml), in the absence (black bars) and the presence (grey bars) of TLR4 inhibitor CLI-095. LPS (10 ng/ml) was used as a positive control. CBA was performed to detect (A) IL-8, (B) TNF-α, (C) IL-6, (D) TNF- α and (E) IL-10 in supernatants from untreated and mucin-treated moDCs.


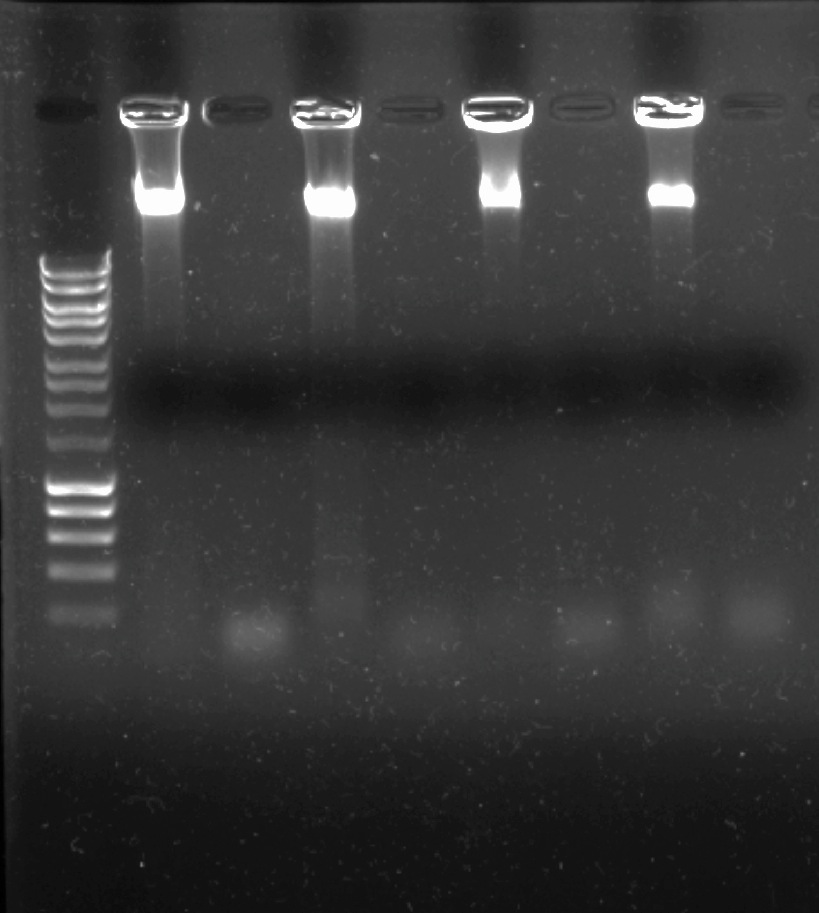


**Mucin SI SI LI LI**

**DNAse -** + **-** +

10000 bp

1000 bp

**Figure S3. Confirmation of DNAse digestion of mucin preparations.** Mouse small intestinal (SI) and large intestinal (LI) mucin preps were treated with DNAse and loaded in a 0.7% agarose gel. Loading order is indicated above the gel (absence of DNAse = -, presence of DNAse = +).

**A**

**B**

**C**

**Figure S4. Mouse intestinal mucin glycopeptides induce moDC activation markers in a TLR4 independent manner.** moDCs were treated with small intestinal (SI), large intestinal (LI) mucins (50 μg/ml) and mucin glycopeptides (50 μg/ml) in the absence (black bars) and the presence (grey bars) of TLR4 inhibitor CLI-095. LPS (10 ng/ml) was used as a control.  **(A)** Detection of IL-8 in supernatants from moDCs either untreated, or treated with mucin glycopeptides (SI GP and LI GP) by ELISA. Results are from 6 independent experiments and statistical significance was assessed using Kruskal Wallis test followed by Dunn’s multiple comparison test (*p<0.05 and **p<0.01). Percentages of **(B)** CD83^+^ and **(C)** CD86^+^ moDCs either untreated or treated with mucin glycopeptides (SI GP and LI GP). Results are from 6 independent experiments and statistical significance was assessed using Kruskal Wallis test followed by Dunn’s multiple comparison test (**p<0.01, ***p<0.001 and ****p<0.0001).

| 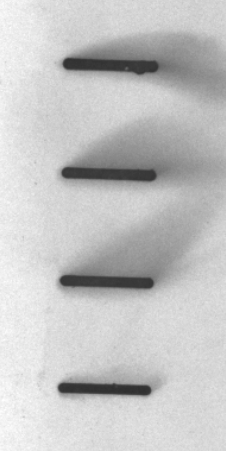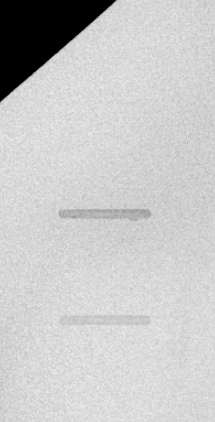 **Schiff’s Muc2**  **Mucin**  Untreated  Oxidized |
| --- |

**Figure S5. Confirmation of mucin oxidation via Schiff’s staining.** To confirm mucin oxidation by periodate, equivalent concentrations of untreated and oxidized mucin were loaded onto a slot blot. Oxidation was detected by Schiff’s reagent (Left blot) and presence of Muc2 was confirmed using an anti-Muc2 antiserum (Right blot).

**MAL II SNA**

**Mucin**

**Untreated**

**Treated**


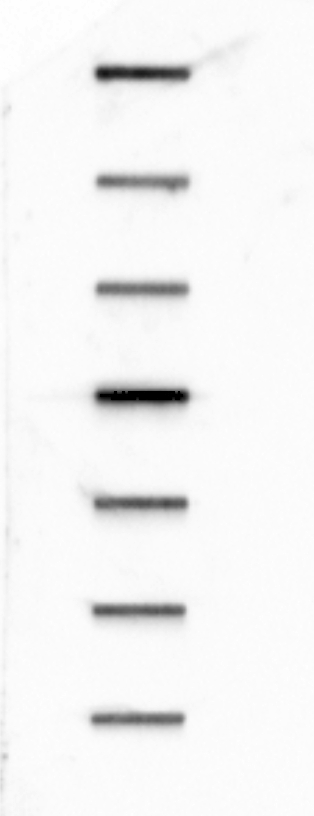

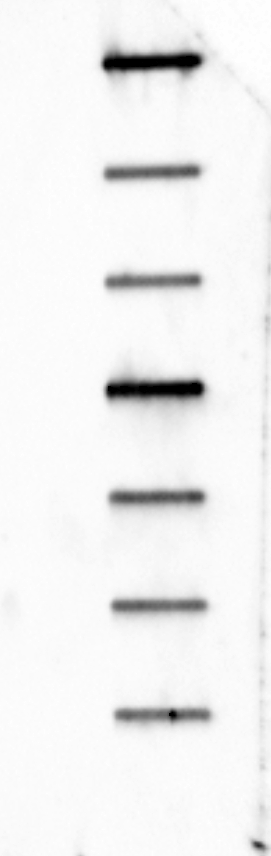


Figure S6. Confirmation of sialic acid reduction from mucins via lectin staining. Mucin digested overnight with neuraminidase was loaded in a slot blot and stained with lectins MAL II (to detect α-2,3-linked sialic acid) and SNA (to detect α-2,6-linked sialic acid). Untreated mucin was loaded as a control.

A

B

**Figure S7. Mucin treatment of moDCs promotes recruit of neutrophil-like cells in a TLR4-independent manner.** (**A**) Transmigration of differentiated neutrophil-like cells HL60 was evaluated in transmigrations assays, exposed to supernatants from moDCs, which were untreated, treated with large intestinal mucin (10 μg/ml), with and without the TLR4 inhibitor CLI-095. Transmigration is expressed as a percentage of migrated cells with respect to untreated moDCs in the presence of no TLR4 inhibitor. Results are from 6 independent experiments and statistical significance was assessed using one-way ANOVA followed by Dunnet’s multiple comparison test. (**B**) Transmigration of primary human neutrophils was similarly evaluated with supernatant from untreated moDC, or moDC treated with 10μg/ml mucin for 4 hours, with and without the TLR4 inhibitor CLI-095. n= 2 independent experiments.

**A**

**B**

**Figure S8. Mouse intestinal mucin does not prevent LPS-induced IL-8 production and DC activation.** moDCs were treated with large intestinal mucin (50μg/ml), LPS (10ng/ml) or both **(A)** Detection of IL-8 in supernatants from moDCs either untreated or treated with mucin and LPS by ELISA. Results are from 4 independent experiments and statistical significance was assessed using Kruskal Wallis test followed by Dunn’s multiple comparison test. **(B)** Percentages of CD86^+^ moDCs either untreated or treated with mucin and LPS. Results are from 2 independent experiments and statistical significance was assessed using Kruskal Wallis test followed by Dunn’s multiple comparison test.

**A**

**B**

**C**

**Figure S9. Synthetic sialic acid does not prevent LPS-induced IL-8 production and DC activation.** moDCs were treated with LPS (10ng/ml) in the in the absence (black bars) and the presence (grey bars) of synthetic sialic acid (100 μg/ml) **(A)** Detection of IL-8 in supernatants from moDCs either untreated or treated with LPS by ELISA. Results are from 4 independent experiments and statistical significance was assessed using Kruskal Wallis test followed by Dunn’s multiple comparison test. Percentages of **(B)** CD83^+^ and **(C)** CD86^+^ moDCs either untreated or treated with LPS. Results are from 4 independent experiments and statistical significance was assessed using Kruskal Wallis test followed by Dunn’s multiple comparison test.

| Gene | Fold change |
| --- | --- |
| *CCR2* | **2.5108** |
| *CD1B* | **2.691** |
| *CD8A* | **2.0823** |
| *CD80* | **2.2786** |
| *CXCR1* | **3.6254** |
| *FLT3LG* | **-2.2066** |
| *ICAM1* | **2.359** |
| *ICAM2* | **2.2163** |
| *IL-8* | **2.0113** |
| *ITGAM* | **2.5636** |

Table S1. Human secreted mucin-induced changes in gene expression of human monocyte-derived DC. PCR arrays using RNA from untreated and human mucin-treated (2 μg/ml) moDC were performed. CT values were normalized with respect to housekeeping genes and fold change between treated vs untreated DC was calculated using Qiagen software. Fold change higher than 2 are shown (1 independent experiment using moDCs differentiated from 1 human donor).

| Identified protein | Accession Number | Total Spectrum Count |
| --- | --- | --- |
| Mucin-2 | MUC2_MOUSE | 40 |
| Protein Fcgbp | E9Q0B5_MOUSE (+1) | 30 |
| Beta-globin | A8DUK4_MOUSE | 17 |
| Histone H4 | H4_MOUSE | 15 |
| Galectin-2 | LEG2_MOUSE | 12 |
| Actin, aortic smooth muscle | ACTA_MOUSE (+1) | 12 |
| Agrin | M0QWP1_MOUSE | 11 |
| Keratin, type I cytoskeletal | K1C19_MOUSE | 11 |
| Isoform 2 of Myosin-11 | MYH11_MOUSE | 7 |
| Serine/arginine-rich splicing factor 7 | SRSF7_MOUSE | 7 |

Table S2. Identified proteins in mouse small intestinal mucin preparation by tandem mass spectrometry. Peptides identified by mass spectrometry were analyzed using the Mascot search engine against the mouse Uniprot database. The total number of spectra obtained for each protein is reported using the Scaffold proteome analysis software (setting a peptide threshold of 95%, top 10 proteins shown).

| Identified protein | Accession Number | Total Spectrum Count |
| --- | --- | --- |
| Mucin-2 | MUC2_MOUSE | 188 |
| Protein Fcgbp | E9Q0B5_MOUSE (+1) | 67 |
| Histone H4 | H4_MOUSE | 25 |
| Prothymosin alpha | PTMA_MOUSE | 25 |
| Isoform 4 of Deleted in malignant brain tumors 1 protein | DMBT1_MOUSE (+1) | 13 |
| Keratin, type I cytoskeletal 19 | K1C19_MOUSE | 13 |
| Actin, aortic smooth muscle | ACTA_MOUSE (+1) | 12 |
| Beta-globin | A8DUK4_MOUSE | 11 |
| Carbonic anhydrase 1 | CAH1_MOUSE | 9 |
| Histone H2B type 2-B | H2B2B_MOUSE | 6 |

Table S3. Identified proteins in mouse large intestinal mucin preparation by tandem mass spectrometry. Peptides identified by mass spectrometry were analyzed using the Mascot search engine against the mouse Uniprot database. The total number of spectra obtained for each protein is reported using the Scaffold proteome analysis software (setting a peptide threshold of 95%, top 10 proteins shown).

| Sample | Molar mass of the reduced original sample | Molar mass of trypsin-digested sample |
| --- | --- | --- |
| Small Intestine | 4.50 (±0.145%) MDa | 0.73 (±0.047%) MDa |
| Large intestine | 5.96 (±0.302%) MDa | 1.67 (±0.038%) MDa |

**Table S4. Characterization of DNA-free mouse mucin glycopeptides.** Small and large intestine mucin preparations treated with DNAse, reduced with DTT and trypsin-digested were further analyzed by SEC-MALLS. Mucin molar mass and concentration was measured by SEC-MALLS and the data were analyzed using Astra software.
